# The Interaction Pattern between a Homology Model of 40S Ribosomal S9 Protein of *Rhizoctonia solani* and 1-Hydroxyphenaize by Docking Study

**DOI:** 10.1155/2014/682946

**Published:** 2014-04-22

**Authors:** Seema Dharni, Sanchita, Abdul Samad, Ashok Sharma, Dharani Dhar Patra

**Affiliations:** ^1^Agronomy and Soil Science Division, CSIR-Central Institute of Medicinal and Aromatic Plants, Lucknow 226015, India; ^2^Biotechnology Division, CSIR-Central Institute of Medicinal and Aromatic Plants, Lucknow 226015, India; ^3^Crop Protection Division, CSIR-Central Institute of Medicinal and Aromatic Plants, Lucknow 226015, India

## Abstract

1-Hydroxyphenazine (1-OH-PHZ), a natural product from *Pseudomonas aeruginosa* strain SD12, was earlier reported to have potent antifungal activity against *Rhizoctonia solani*. In the present work, the antifungal activity of 1-OH-PHZ on 40S ribosomal S9 protein was validated by molecular docking approach. 1-OH-PHZ showed interaction with two polar contacts with residues, Arg69 and Phe19, which inhibits the synthesis of fungal protein. Our study reveals that 1-OH-PHZ can be a potent inhibitor of 40S ribosomal S9 protein of *R. solani* that may be a promising approach for the management of fungal diseases.

## 1. Introduction

Microorganisms are capable of synthesizing versatile chemical structures with diverse biological activities beyond the scope of synthetic organic chemistry [1] and can be directly used as fungicide products or as lead molecules for designing of novel synthetic products. Phenazine constitutes a large group of nitrogen containing heterocyclic compounds that are substituted at different sites of the core ring system and therefore displays a wide range of structural derivatives and are remarkable for the multiplex mechanism of their biological activities. More than 100 different phenazine structural derivatives have been identified and over 6,000 compounds that contain phenazine as a central moiety have been synthesizedand 100 biologically active (antibacterial, antifungal, antiviral, and antitumor) phenazines from natural origin are known to date, synthesized mainly by* Pseudomonas* and* Streptomyces* species [2–4]. Fluorescent pseudomonades such as* Pseudomonas fluorescens *2–79*, Pseudomonas chlororaphis *(previously named* P. aureofaciens *30–84) [5], and* Pseudomonas aeruginosa* are the best studied phenazine producers [2].

Phenazines are site-specific inhibitors which target individual sites within the fungal cells or multisite inhibitors which target different sites in each fungal cell. Phenazine acts in three ways; one of the mechanisms is underlying those which inhibit energy production by blocking SH-groups, the glycolysis/citrate cycle, or the respiratory chain. Second that inhibits biosynthesis of proteins, nucleic acids, cells walls, and membrane lipids, or interfere with mitosis, and third which induces indirect effects which change host/pathogen interactions [6]. The enzymes involved in any of the above-mentioned processes can be considered as a target receptor and the metabolite as ligand. Molecular docking studies can also be performed for microbial fungicides to validate their inhibiting properties.

Clofazimine is a synthetic phenazine analogue belonging to the riminophenazines group of compounds which was originally discovered in lichens [2, 7] and another phenazine, bis (phenazine-1-carboxamide), acts as a potent cytotoxin and represents an interesting class of dual topoisomerase I/II directed anticancer activity [8]. The highlight of biological significance of phenazines is their ability to act as broad-spectrum antimicrobial, antiparasite, antimalarial, and antifungal agents affecting a vast range of organisms [2, 9, 10]. Inhibition of DNA-dependent RNA synthesis in the absence of detected DNA intercalation has been observed for lomofungin during elongation, which has been shown to block the transcription complex at the initiation state as well as during elongation [11].

Phenyl amides (PA) fungicides affect nucleic acids synthesis by inhibiting the activity of the RNA polymerase I system which interferes with nucleic acid synthesis, thus blocking rRNA synthesis [12]. Phenylpyrrole fungicidal ingredient, fludioxonil, (4-(2,2-difluoro-1,3-benzodioxil-4-yl)-1H-pyrrole-3-carbonitrile), produced by* Pseudomonas pyrrocinia, *revealed inhibition of spore germination and germ tube elongation [13]. Tubericidin produced by* Streptomyces violaceoniger*, was highly active against* Phytophthora capsici* and* Rhizoctonia solani*. Tubericidin interferes the nucleic acid synthesis, including* de novo* purine synthesis, rRNA processing, and tRNA methylation [14]. The derivatives of phenazine—antibiotics iodine, muxin, and pyocyanin—are capable of interacting with DNA/RNA either by blocking the template (DNA intercalation), binding to RNA polymerase, or binding to a ribonucleoside 5′-triphosphate [15]. A new phenazine-1-carboxylic acid phenylamide (PCA-1-P) exhibited substantial growth retardation of three gram-positive and the strong inhibitory activity of PCA-1-P derivatives towards the RNA synthesis* in vitro* T7-RNA-polymerase [16].

In this study we have described the mode of action of 1-OH-PHZ inhibiting the 40S ribosomal protein S9 of* R. solani *through docking approaches. The 40S ribosomal protein S9 plays a central role in the initiation factors considered to be a primary rRNA-binding protein that facilitates scanning of messenger RNAs and initiation of protein synthesis. The ribosomal protein S9 is an essential protein located at the entrance tunnel of the mRNA into the ribosome and plays an accurate role in decoding. Lindström and Zhang reported that ribosomal protein S9 is required for normal cell growth and proliferation, as depletion of S9 resulted in decreased protein synthesis which is associated with G_1_ cell cycle arrest [17]. A recent study has been shown that ribosomal protein S9 is located at the entrance tunnel of mRNA in the ribosomes and is involved in regulation of mRNA translation, possibly translation termination [18]. It will be of considerable interest in the future to further find out a specific antifungal agent targeting on inhibition of the translation step which has the effect of blocking protein production and ultimately its function.

## 2. Materials and Methods

### 2.1. Bacterial Strain

SD12 was isolated from metal polluted soil as previously described by Dharni et al. [19]. The strain was also deposited at Microbial Type Culture Collection (MTCC), IMTECH, Chandigarh, India (http://mtcc.imtech.res.in/), with accession number 106439.

### 2.2. 1-Hydroxyphenaizne (1-OH-PHZ)

As described previously by Dharni et al. [19], 1-OH-PHZ was purified from the culture supernatant of* P.aeruginosa* SD12 by using stepwise gradient vacuum liquid chromatography.

### 2.3. Nucleotide Sequence Accession Number

The nucleotide sequence of 16S rRNA of strain SD-12 has been reported in the GenBank database of NCBI (http://www.ncbi.nlm.nih.gov/) having the accession number HQ268805.

### 2.4. Structure Prediction of Protein

The three-dimensional structure of 40S ribosomal protein S9 of* Rhizoctonia solani* is not available in any database. For deducing the structure, the protein sequence (176 amino acid) was obtained from NCBI database (acc. no. ABE68880) and uploaded in FASTA format in SWISS-MODEL via the ExPASY web server [20]. To obtain the closest match BLAST of query protein sequence was performed which searched against Protein Data Bank (PDB), http://www.rcsb.org/pdb/home/home.do [21]. The modeled structure of the protein was submitted to the PMDB database, http://mi.caspur.it/PMDB/main.php.

### 2.5. Structure Validation

After the model generation, its quality assessment was done, based on both geometric and energetic aspects. The SWISS PDB Viewer was used for minimizing the energy of modeled protein [22]. The stereochemical properties of obtained protein model were checked using RAMPAGE server [23]. The ramachandran plot provided the residue position in particular segments based on *φ* and *ψ* angles between N-C_*α*_ and C_*α*_-C atoms of residues.

### 2.6. Ligand Structure

For docking study, 1-hydroxyphenazine was taken as a ligand compound. The structure of this compound was retrieved from Pubchem database maintained at NCBI, http://pubchem.ncbi.nlm.nih.gov/. The three-dimensional structure of ligand was converted from mol to pdb format to input into the Autodock. This conversion was done through Open Babel tool, http://sourceforge.net/projects/openbabel/.

### 2.7. Molecular Docking

The docking of ligand (1-OH-PHZ) with modeled 40S ribosomal S9 protein was performed by Autodock 4.2 using genetic algorithm approach [24]. The grid box dimension of 0.375 Å was selected in protein for docking with ligand. The blind docking approach was acquired as this gives good results in substrate binding site prediction [25]. In this approach, the full flexible ligand was used for docking while keeping the protein in a fixed orientation in space. The negative and lower value of binding energy as well as more numbers of hydrogen bonds showed favored binding between ligand and target.

## 3. Results and Discussion

### 3.1. 1-Hydroxyhenazine (1-OH-PHZ)

**Figure 1:**
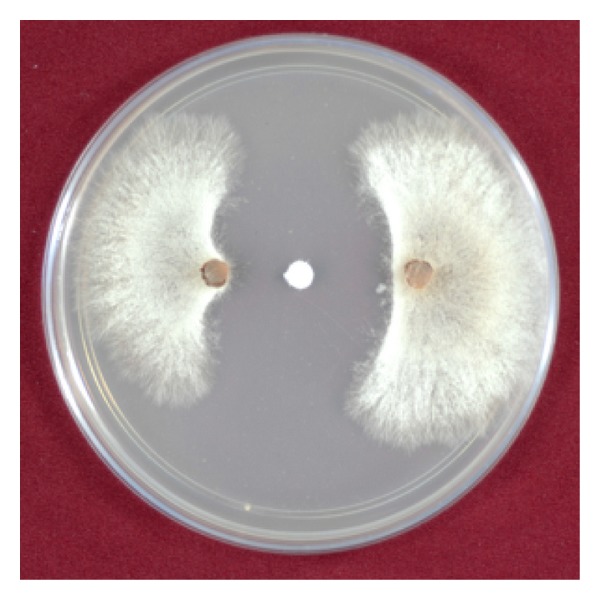
Antifungal activity of 1-hydroxyphenazine at 40 *μ*g/disc against* Rhizoctonia solani*.

Our previous studies demonstrated that 1-OH-PHZ showed antifungal activity against* R. solani*, a soilborne pathogen, at 40 *μ*g/disc (Figure 1) [19]. 1-OH-PHZ earlier isolated from* P. aeruginosa* TISTR 781 [26] was reported to inhibit* Escherichia coli* and* Xanthomonas campestris pv. vesicatoria*. Recently, phenazine-1-carboxylic acid (PCA) produced by* Pseudomonas* sp. M18G is being marketed as* Shenquinmycin* in China, which has gained a Pesticide Registration Certification (*Fusarium oxysporum*) issued by the Chinese Ministry of Agriculture, owing to its high efficiency against various phytopathogens, low toxicity, and good environmental compatibility. It is proved as an effective agent for the biocontrol of withering of watermelon sprout (code) and piemintoepidemic disease (*Pythium capsici*) [27, 28]. 1-OH-PHZ was earlier isolated from* P. aeruginosa* TISTR 781 and known to inhibit* Escherichia coli* and* Xanthomonas campestris pv*.* vesicatoria* [26]. N-acrylamides of 9-substituted phenazine-1-carboxylic acids (PCA) have been reported as strong inhibitors of RNA synthesis [29, 30].

### 3.2. Secondary Structure Prediction

**Figure 2:**
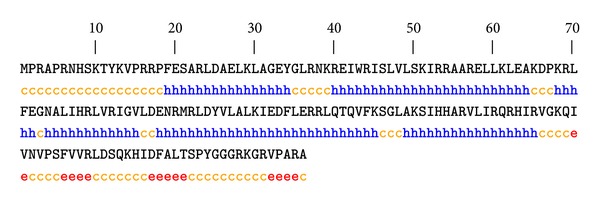
Secondary structure of modeled protein of* Rhizoctonia solani*.

GOR4 server [12] was used for secondary structure prediction of modeled protein. Helix, sheet, and coils were the secondary structures found to occur in the protein. GOR4 server reveals 58.52% residues in *α* helices, 8.52% residues in *β* sheet, and 32.95% residues in random coils (Figure 2). The results revealed that *α* helices contributed more as compared to other structures. These structures were joined together to form a three-dimensional structure of protein. The secondary structures were predicted by using default parameters. The results have been validated through Chou Fasman servers [31].

### 3.3. Homology Modeling of 40S Ribosomal S9 Protein

**Figure 3:**
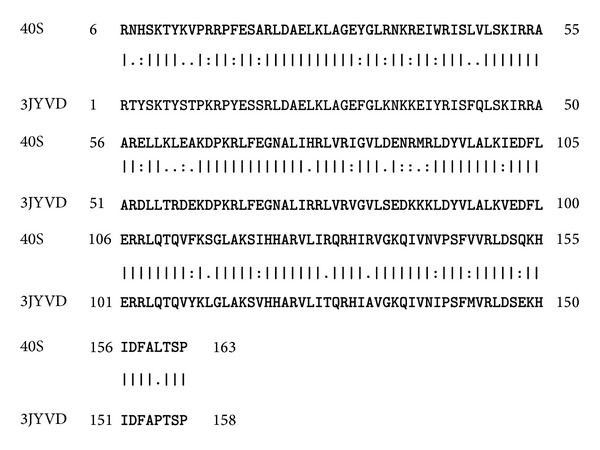
BLAST sequence alignment of 40S ribosomal protein S9 and 3JYV using BLOSUM62 matrix. The highly aligned regions are shown in (∣).

**Figure 4:**
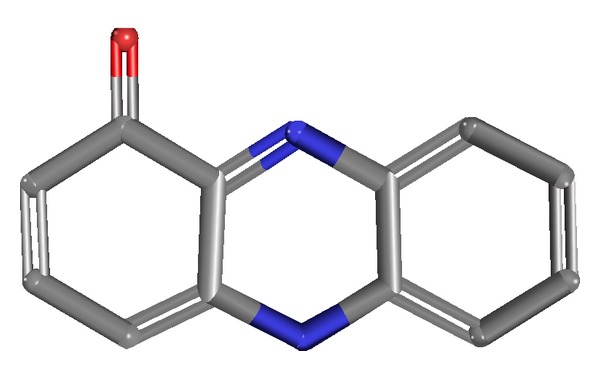
Schematic representation of 1-hydroxyphenazine.

**Figure 5:**
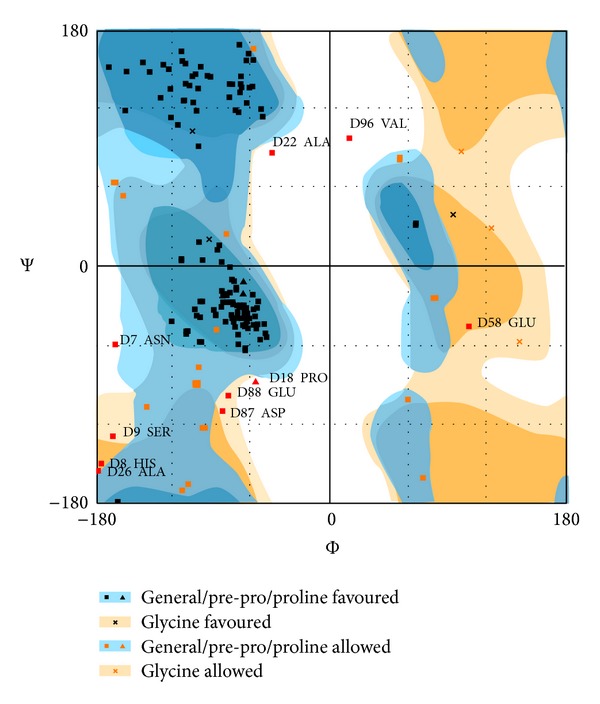
Modeled structure of 40S ribosomal S9 protein.

**Figure 6:**
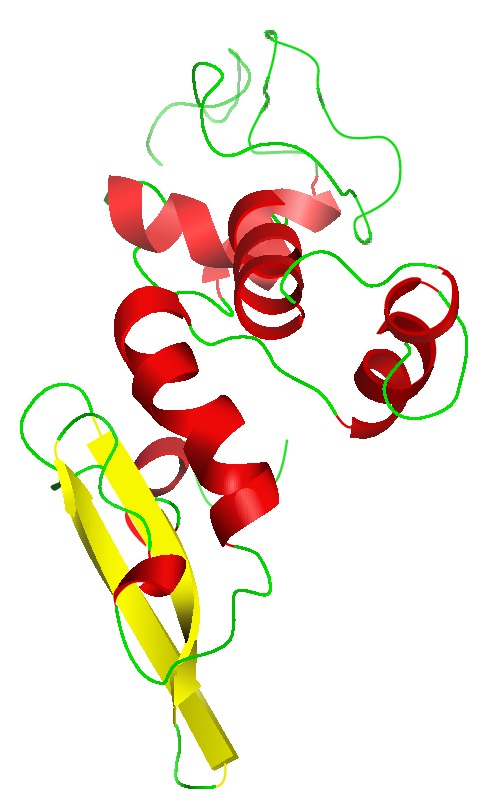
Ramachandran map of 40S ribosomal ligand moleS9 protein of* Rhizoctonia solani*.

40S ribosomal S9 protein is an important target responsible for fungal growth [32]. To predict the structure of this protein, homology modeling approach was performed. The query sequence of target protein was found homologous to the known structure of protein of the same family of* Thermomyces lanuginosus. *After BLAST search the high resolution crystal structure of homologous protein having PDB id 3JYV was considered as template for comparative modeling. The query and template sequences showed 78% identity and 6*e* − 87 Evalue in sequence alignment and were taken as template for homology modeling (Figure 3). The structure was modeled on automated mode of SWISS-MODEL workspace, http://swissmodel.expasy.org/, with its default parameters. The residues ranging between 6 and 163 participated in homology modeling. The total energy −5238.173 KJ/mol of the model was converted to −7587.487 KJ/mol after minimizing the energy through SWISS PDB Viewer. The final predicted 3D structure of the protein was represented through Pymol viewer, http://www.pymol.org/, (Figure 4). Alpha helices are shown in green color and antiparallel beta sheet in red color in the structure (Figure 5). There are many loop structures that help in connecting the secondary structures together. The model was submitted to the PMDB database and it can be downloaded using id “PM0078917.” The modeled structure of protein was evaluated with ramachandran plot generated by Rampage server, http://mordred.bioc.cam.ac.uk/~rapper/rampage.php, and fulfilled the criterion of proper distribution of residues in different regions** (** Figure 6) [21]. 82.1% and 11.5% of the residues were present in favored and allowed regions, respectively.

### 3.4. Docking Studies

**Figure 7:**
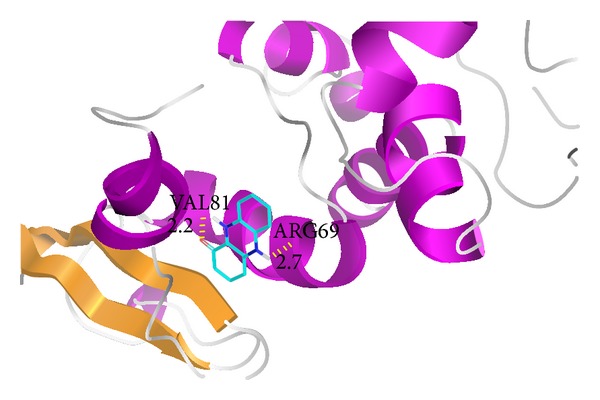
Docking of 1-OH-PHZ with 40S ribosomal S9 protein.

The Autodock 4.2 program was used for the molecular docking analysis [24]. The three-dimensional structure of 1-OH-PHZ was considered as ligand molecule (Figure 7). The interaction of this ligand with modeled protein was performed. The Lamarckian genetic algorithm was used in Autodock to perform the automated molecular dockings [33] and default parameters were used. In the process a large grid map was created by AutoGrid to cover the whole surface of protein. By performing the rigid docking, a total of 10 conformations of interaction were obtained having variation in their energies, that is, free energy of binding, predicted inhibition constant, and ligand efficiency (Table 1). From all the docking conformations of ligand, conformation 3 was selected for having −5.84 kcal/mol binding energy and 1 hydrogen bond. The ligand, 1-OH-PHZ, was found to be bound with two residues of protein by polar contacts. These contacts are present at VAL81 and Arg69 positions of the protein.

## 4. Conclusion

Our docking results showed that 1-OH-PHZ obtained from* Pseudomonas aeruginosa *SD12 may be a potent inhibitor against* Rhizoctonia solani*. A homology model of 40S ribosomal S9 protein of* R. solani* was built and validated through ramachandran plot. Validation by the software showed that the homology model energy score is similar to the crystal structure of the template. SWISS model was used to develop a reliable model for performing the docking study. This docking study of the 40S ribosomal S9 protein showed that out of 10 docked conformations, the 3rd conformation was the best because it has comparatively lower binding energy as well as hydrogen bonding. These observations may be of great help in the QSAR based designing of fungicides from a very common, inexpensive, and nontoxic natural product, which is safe for humans, animals, and environment. This study will also facilitate the understanding of the structural and functional basis of ligand binding to the protein for further research.

## Figures and Tables

**Table 1:** Docking results and *K*_*i*_ values for each conformation studied.

Conformations	Free energy of binding (kcal/mol)	Predicted inhibition constant, *K*_*i*_ (uM)	Ligand efficiency	Hydrogen bonding	Residues
1	−5.44	103.69	−0.36	1	LEU76
2	−5.94	44.45	−0.4	No	
3	−5.84	52.24	−0.39	1	ARG69
4	−5.44	103.62	−0.36	1	LEU76
5	−5.94	44.44	−0.4	No	
6	−5.84	52.06	−0.39	1	ARG69
7	−5.44	103.67	−0.36	1	LEU76
8	−5.84	52.12	−0.39	1	ARG69
9	−5.44	103.32	−0.36	1	LEU76
10	−5.84	52.12	−0.39	1	ARG69
